# Autotetraploid *Coffea canephora* and Auto-Alloctaploid *Coffea arabica* From *In Vitro* Chromosome Set Doubling: New Germplasms for *Coffea*

**DOI:** 10.3389/fpls.2020.00154

**Published:** 2020-03-04

**Authors:** Lucimara Ribeiro Venial, Maria Andréia Corrêa Mendonça, Paulo Marcos Amaral-Silva, Guilherme Bravim Canal, Ana Beatriz Rocha de Jesus Passos, Adésio Ferreira, Taís Cristina Bastos Soares, Wellington Ronildo Clarindo

**Affiliations:** ^1^ Laboratório de Citogenética e Cultura de Tecidos Vegetais, Centro de Ciências Agrárias e Engenharias, Universidade Federal do Espírito Santo, Alegre, Brazil; ^2^ Laboratório de Biotecnologia, Instituto Federal Goiano—Campus Rio Verde, Rio Verde, Brazil; ^3^ Laboratório de Biometria, Centro de Ciências Agrárias e Engenharias, Universidade Federal do Espírito Santo, Alegre, Brazil; ^4^ Laboratório de Bioquímica e Biologia Molecular, Departamento de Farmácia e Nutrição, Centro de Ciências Exatas, Naturais e da Saúde, Universidade Federal do Espírito Santo, Alegre, Brazil; ^5^ Laboratório de Citogenética e Citometria, Departamento de Biologia Geral, Centro de Ciências Biológicas e da Saúde, Universidade Federal de Viçosa, Viçosa, Brazil

**Keywords:** coffee, polyploidy, plant tissue culture, flow cytometry, cytogenetics, whole-genome duplication

## Abstract

Polyploidy is more than two chromosomal sets per nucleus, as the allotetraploid *Coffea arabica*. Due to allotetraploidy, *C. arabica* shows different phenotypes compare to diploid *Coffea* species, highlighting by beverage quality produced from its grains. Looking for the possibility of new phenotypes coupled with economic feature, considerable progress since 60’s was reached for synthetic chromosome set doubling (CSD) *in vitro*, involving especially the antitubulin compounds, biological material, and used tissue culture pathway as the indirect somatic embryogenesis (ISE). Here, we aimed to regenerate autotetraploid and auto-alloctaploid plantlets of *Coffea canephora* and *C. arabica*, respectively, from a novel *in vitro* CSD procedure for *Coffea*. Exploring the ISE pathway, we treated the cellular aggregate suspensions (CAS) with 0.0 (control), 0.5, 1.5, or 2.5 mM of colchicine solution for 48, 72, or 96 h and maintained in liquid medium under constant orbital shaking. After transferring the CAS to semisolid media for somatic embryo regeneration, we considered it as cellular mass. Mature cotyledonary somatic embryos were only regenerated from cellular masses treated with 2.5 mM/48 h and 2.5 mM/72 h for *C. canephora* and with 0.5 mM/48 h for *C. arabica*. Evaluating the DNA ploidy level and the chromosome counting revealed that 36 (34.9%) plantlets of *C. canephora* were autotetraploids (4C = 2.86 pg, 2n = 4x = 44) and 61 (21.1%) of *C. arabica* were auto-alloctaploids (4C = 5.24 pg, 2n = 8x = 88). The CSD procedure, exploring the CAS proliferation and ISE pathway, promoted whole-genome duplication and resulted in a relatively high number of solid polyploids of both *Coffea* species. Due to distinct responses, DNA sequence fidelity (genetic) and global level of 5-methylcytosine (epigenetic) were evaluated. We observed that the increase of 5-methylcytosine levels was associated with somatic embryo regeneration from cells showing DNA sequence fidelity for the tested SSR primers. In conclusion, the adopted procedure for *in vitro* CSD is reproducible for induction, regeneration and propagation of *Coffea* polyploids and potentially other shrubbery and woody species. In view of the novelty of this procedure to generate new germplasm, we show the key issues and the steps of the CSD procedure.

## Highlights

We established a novel chromosome set doubling procedure for *Coffea* treating cellular aggregate suspensions with colchicine. From this procedure, new *Coffea canephora* autotetraploids and *Coffea arabica* auto-alloctaploids were regenerated.

## Introduction

Polyploidization leads to more than two complete chromosome sets per nucleus in a cell, naturally occurring through autopolyploidy or allopolyploidy (Stebbins, 1947). As a result of “omic” changes (genomic, epigenomic, transcriptomic, and metabolomic), polyploids may exhibit new physiological, morphological and reproductive phenotypes and/or traits (Sattler et al., 2016; Li et al., 2019; Iannicelli et al., 2020). Because of this, polyploidy has been considered an important trigger in plant diversification and evolution (Soltis et al., 2009; Iannicelli et al., 2020), including the saltational speciation (Mallet, 2007; Iannicelli et al., 2020).

The impact of natural polyploidy on plant diversity and evolution, but also in worldwide economy and breeding programs, have inspired several research groups to establish different strategies for synthetic polyploidization through chromosome set doubling (CSD). In an agronomic scenario, the *ex vitro* and *in vitro* procedures to induce synthetic polyploidy lead to new and/or improved germplasms, enhancing the breeding programs of crop, ornamental, medicinal and forest species (Dhooghe et al., 2011; Sattler et al., 2016; Iannicelli et al., 2020). Synthetic polyploids have been obtained mainly from CSD in *in vitro* environments following Murashige and Nakano (1966), under controlled physical and chemical conditions. Biological material showing proliferative cells, mainly shoot tips, is exposed to the antitubulinic agent (e.g., colchicine, oryzalin, trifluralin, amiprophos-methyl) added to the tissue culture medium. These compounds prevent the mitotic or meiotic spindle microtubule (fuse) formation through binding to α- and/or β-tubulin (Planchais et al., 2000). Due to this cytotoxic effect, the sister chromatids (mitotic anaphase, meiotic anaphase II) and homologues chromosomes (meiotic anaphase I) disjunction as well as the cytokinesis do not occur, resulting in cells with duplicated chromosome set. Regarding the *in vitro* strategies, the chromosome set has been successfully duplicated for trees and shrubs, like *Acacia dealbata* Link., *Acacia mangium* Willd. (Blakesley et al., 2002), *Platanus acerifolia* (Ait.) Willd. (Liu et al., 2007), *Jatropha curcas* L. (de Oliveira et al., 2013), *Ziziphus jujuba* Mill. (Shi et al., 2015), *Eriobotrya japonica* (Thunb.) Lindl. (Blasco et al., 2015), allotriploid “Híbrido de Timor” (*Coffea canephora* Pierre ex A. Froehner *x Coffea arabica* L., Sanglard et al., 2017), *Eucalyptus grandis* W. Hill ex Maiden, *Eucalyptus urophylla* S. T. Blake, *Eucalyptus benthamii* Maiden & Cambage, and homoploid *E. urophylla x E. grandis* (Silva et al., 2019).

In order to expand the applicability, improvements have been made to solve the main bottlenecks of the *in vitro* CSD procedure: low rate of solid polyploids and high rate of mixoploids, as well as propagule mortality. Nowadays, the more promising *in vitro* procedure associates the indirect somatic embryogenesis (ISE) pathway with the antitubulin agent treatment. This *in vitro* pathway is based on somatic embryo recovery—the possibility of regenerating a plantlet from a single cell (Stewart et al., 1958)—which maximizes the occurrence of only solid polyploids from CSD exploring the ISE (Wu and Mooney, 2002; Dutt et al., 2010; Acanda et al., 2015; Sanglard et al., 2017).

Pro-embryogenic cells of friable calli in semisolid medium (Wu and Mooney, 2002; Petersen et al., 2003; Zhang et al., 2007; Sanglard et al., 2017) or of cellular aggregate suspensions (CAS) in liquid medium (Dutt et al., 2010; Acanda et al., 2015) have been exposed to different antitubulin agents for different times and concentrations. CSD was performed from semisolid system for *Spathiphyllum wallisii* Regel (Eeckhaut et al., 2004), *Citrus* L. (Wu and Mooney, 2002; Petersen et al., 2003; Zhang et al., 2007), homoploid *Vitis* x *Muscadinia* (Xie et al., 2015), anorthoploid *Coffea* “Híbrido de Timor” (Sanglard et al., 2017), *Lilium distichum* Nakai, and *Lilium cernuum* Komar (Fu et al., 2019). Differently, Dutt et al. [(2010), for *Citrus reticulata* Blanco] and Acanda et al. [(2015), for *Vitis vinifera* L. “Mencía”] conducted the CSD from CAS, which they established from friable calli propagated in liquid medium. CAS show a high cell proliferation rate (van Boxtel and Berthouly, 1996) that is the feedstock for CSD. The gradient of nutrients in the liquid medium is considered another advantage over semisolid system (Dutt et al., 2010), increasing cell contact with the tissue culture compounds and with the antitubulin used for CSD.

Variations in the ISE response have been observed after treatment with antitubulin agents, as the rate of somatic embryos and plantlets (responsive or unresponsive) recovered from the friable calli or CAS (Wu and Mooney, 2002; Zhang et al., 2007; Sanglard et al., 2017). The causes of these variations can be associated with the *in vitro* conditions, the occurrence of somaclonal variation during the ISE pathway and/or the cytotoxic effect of the antitubulin treatment that involves a pulse using different compounds and concentrations (Dhooghe et al., 2011). Thus, genetic (SSR markers) and epigenetic (global methylated cytosine) features are appointed as possible factors that interfere in the somatic embryo and plantlet regeneration (Dutt et al., 2010). Therefore, these aspects should be investigated in order to understand the distinct ISE responses.

Owing to the new genomic and phenomic features of the polyploids in relation to their ancestors, there is great interest to achieve synthetic polyploids. The aim of this work was to establish a new procedure for CSD from CAS of the agronomic relevant *Coffea* species *C. canephora* and *C. arabica*. Additionally, we evaluate the DNA sequence stability (SSR markers) and epigenetic (5-methylcytosine) level to find possible causes for the varying ISE responses. We choose *C. canephora* and *C. arabica* because of the previously established ISE for these species by our research group (Sanglard et al., 2019). Besides, *C. canephora* and *C. arabica* are relevant for the economy of some countries, like Brazil, Vietnam, Colombia, Mexico, Indonesia, India, Guatemala, Uganda, and Ethiopia. The grains produced by *C. arabica* represent 70–75% of the exported coffee for Brazil, and 25–30% by *C. canephora*. Thus, we also expected to provide new *C. canephora* and *C. arabica* polyploid germplasms for future breeding approaches involving selection of the individuals based on their grain production, coffee beverage quality and tolerance and resistance to abiotic and biotic stresses, as well as for crossing with other *Coffea*.

## Materials and Methods

### Plant Material

One *C. canephora* plantlet, which has been propagated in an *in vitro* germplasm bank (Universidade Federal do Espírito Santo, Espírito Santo, Brazil), and one *C. arabica* plant, which has been maintained in a greenhouse bank (Universidade Federal de Viçosa, Minas Gerais, Brazil), were used as explant donor plants. Chromosome number, ploidy level and DNA content of the explant donor plants were confirmed as 2n = 2x = 22 chromosomes and 2C = 1.43 pg for *C. canephora*, and 2n = 4x = 44 chromosomes and 2C = 2.62 pg for *C. arabica*. These *Coffea* crops were chosen due to agronomic and evolutive relevance. *C. canephora* is a diploid species with 2n = 2x = 22 chromosomes and 2C nuclear DNA content of 1.43 pg, and *C. arabica* is a true allotetraploid that possesses 2n = 4x = 44 chromosomes and 2C = 2.62 pg (Sanglard et al., 2019). *C. arabica* is the only polyploid species of the *Coffea* genus, which was probably originated from crossing between the diploid species *Coffea eugenioides* S. Moore and *C. canephora*. Besides the karyotype, the divergences between these species also include the reproductive mechanism (*C. canephora* is allogamous, like the other diploid *Coffea* species, and *C. arabica* is autogamous, Yu et al., 2011), morphological and physiological aspects (Charrier and Berthaud, 1985), and commercial relevance mainly associated to the beverage quality of coffee (Farah and Donangelo, 2006).

### Friable Calli Induction and Cellular Aggregate Suspensions Establishment

We collected and disinfected leaves of *Coffea arabica* according to Sanglard et al. (2019) before inoculation. We excised five leaf fragments of 1cm² from both species and inoculated them in M1 medium (**Table 1**) in 60 × 15 mm Petri dishes. The culture was conducted in the dark at 25 ± 2°C for friable calli induction. After 60 days, 0.5 g of friable calli was transferred to 125 ml Erlenmeyers containing 30 ml of M2 medium (**Table 1**). The Erlenmeyers were maintained in the dark at 25 ± 2°C on a 100 rpm orbital shaker. For establishment of the CAS, the material was subcultured every 15 days into a fresh medium, respecting the 0.5 g of cellular aggregates per Erlenmeyer. All procedures were performed under aseptic conditions.

**Table 1:** Tissue culture media used for friable calli induction (M1), CAS establishment (M2), CSD (M2), somatic embryo (M3 and M4) and plantlet regeneration (M5) of *C. canephora* and *C. arabica*.

Components	Medium				
	M1	M2	M3	M4	M5
MS (Sigma^®^)	2.15 g L^-1^	2.15 g L^-1^	4.30 g L^-1^	4.30 g L^-1^	4.30 g L^-1^
Gamborg’s B5 vitamins	10 ml L^-1^	10 ml L^-1^	10 ml L^-1^	10 ml L^-1^	10 ml L^-1^
Sucrose (Sigma^®^)	30 g L^-1^	30 g L^-1^	30 g L^-1^	30 g L^-1^	30 g L^-1^
L-cysteine (Sigma^®^)	0.08 g L^-1^	0.04 g L^-1^	0.04 g L^-1^	0.04 g L^-1^	0.04 g L^-1^
Malt extract (Sigma^®^)	0.4 g L^-1^	0.4 g L^-1^	0.4 g L^-1^	0.4 g L^-1^	0.4 g L^-1^
Casein hydrolysate (Sigma^®^)	0.1 g L^-1^	0.1 g L^-1^	0.1 g L^-1^	0.1 g L^-1^	0.1 g L^-1^
2,4-D (Sigma^®^)	9.06 µM	9.06 µM	–	–	–
BAP (Sigma^®^)	4.44 µM	4.44 µM	4.44 µM	4.44 µM	–
GA_3_ (Sigma^®^)	–	–	–	–	2.89 µM
Phytagel (Sigma^®^)	2.8 g L^-1^	–	2.8 g L^-1^	2.8 g L^-1^	2.8 g L^-1^
Activated charcoal (Isofar^®^)	–	–	2.0 g L^-1^	4.0 g L^-1^	–
pH	5.6	5.6	5.6	5.6	5.6

MS, Murashige and Skoog (1962); Gamborg’s B5 vitamins, Gamborg et al. (1968); 2,4-D, 2,4-dichlorophenoxyacetic acid; BAP: 6-benzylaminopurine; GA_3_, gibberellic acid. Culture media were sterilized in an autoclave at 121°C and 1.5 atm for 20 min.

### Chromosome Set Doubling and Plantlet Recovering

**Figure 1:**
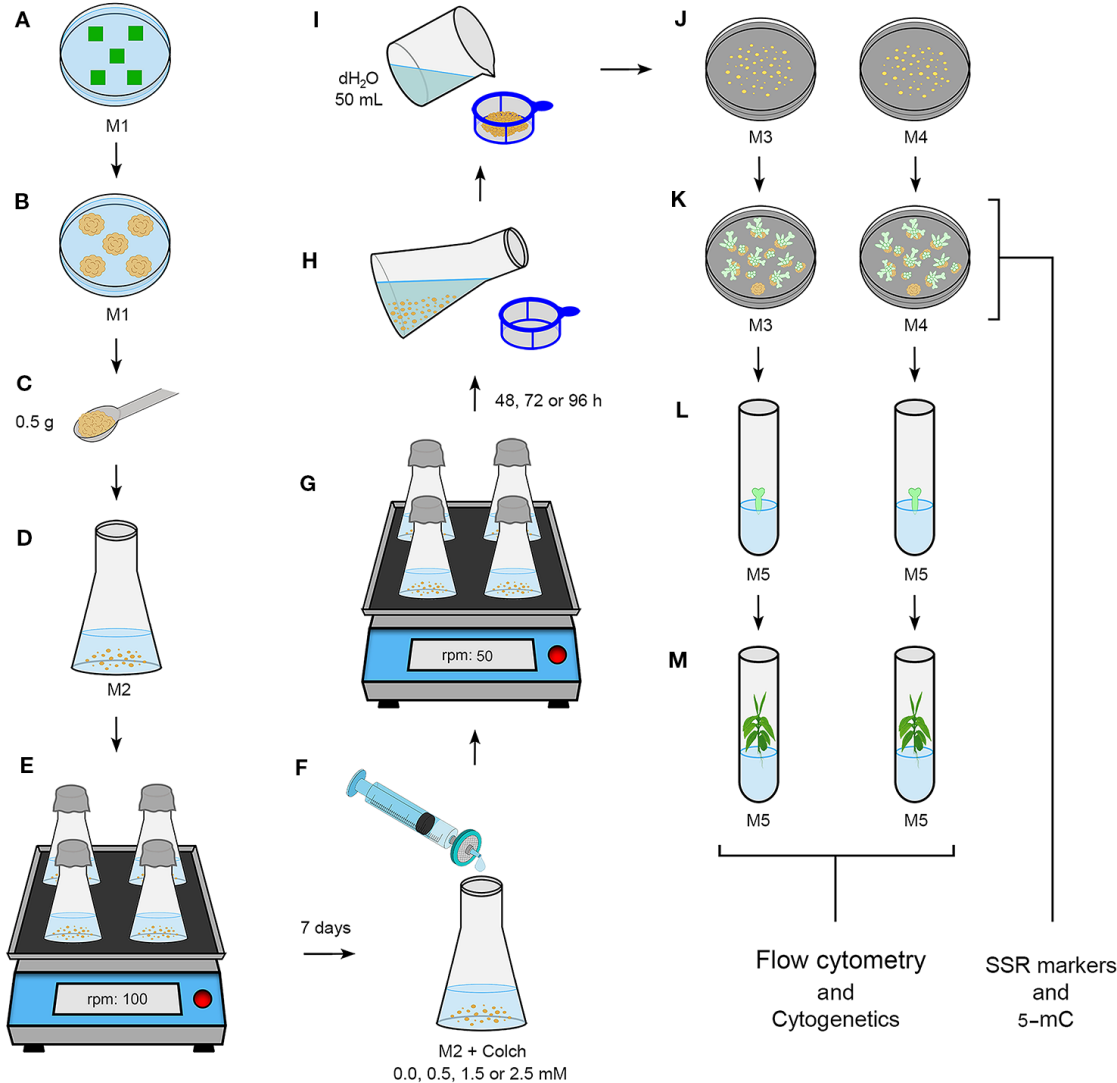
Novel procedure for CSD from CAS treated with colchicine. **(A)** *Coffea* leaf fragment explants (~1 cm²) in M1 medium (**Table 1**). **(B)** Friable calli after ~30 days in M1.** (C, D)** 0.5 g of friable calli in 125 ml Erlenmeyers containing 30 ml of M2 medium (**Table 1**). **(E)** CAS on orbital shaker at 100 rpm. **(F)** Filter sterilized colchicine solution addition at different concentrations after seven days, according growth curve reported for *Coffea* (van Boxtel and Berthouly, 1996). **(G)** CAS under colchicine treatment for distinct times on orbital shaker at 50 rpm. **(H, I)** Careful and continous washing of the CAS with 50 ml sterile dH_2_O. **(J)** Cellular mass (CM) in M3 or M4 medium (**Table 1**). **(K)** CM showing *C. canephora* or *C. arabica* globular, heart, torpedo and cotyledonary embryos in M3 and M4. CM samples of “j” and “l” were collected to evaluate and compare the DNA sequence variability and 5-methylcytosine level. **(L)** MCSE in M5 medium (**Table 1**). For *C. arabica*, these embryos were obtained after 120 days, and for *C. canephora* after 180 days. **(M)** Regenerated plantlets of the two *Coffea* species, of which we excised leaves for DNA ploidy level determination and root meristems for chromosome counting.

After the fourth subculture, the CAS were treated with colchicine, an alkaloid compound isolated from *Colchicum autumnale* L. seeds and bulbs (Planchais et al., 2000). We applied the colchicine treatment 7 days after the fourth subculture, according to growth curves of the *C. canephora* and *C. arabica* CAS (van Boxtel and Berthouly, 1996). For this, we added filter-sterilized colchicine solution to each Erlenmeyer: 0.0 (control), 0.5, 1.5, and 2.5 mM, respectively for each treatment. The CAS were maintained under colchicine treatment in the dark, on a 50 rpm orbital shaker at 25 ± 2°C for 48, 72, and 96 h. One Erlenmeyer referred to each treatment (colchicine/time), adding up to 12 in total. After colchicine exposition, the CAS of each Erlenmeyer was separately filtered through a 40 µm cell strainer (BD Falcon™), and carefully washed with at least 50 ml of autoclaved dH_2_O for residual elimination of colchicine (**Figure 1**). Cellular aggregates of each Erlenmeyer were subdivided in 60 × 15 mm Petri dishes containing M3 or M4 medium (**Table 1**). After transferring the CAS to semisolid medium, the cellular aggregates were denominated as cellular mass. After regeneration of somatic embryos, only the mature cotyledonary somatic embryos (MCSE) were transferred to tubes containing M5 medium for germination (**Table 1**).

### Ploidy Level of the Recovered Plantlets

Initially, we determined the DNA ploidy level of the regenerated *C. canephora* and *C. arabica* plantlets from nuclei suspensions extracted from leaf fragments (~1 cm^2^) by chopping (Galbraith et al., 1983) and staining according to Otto (1990) and Praça-Fontes et al. (2011). Nuclei suspensions obtained from leaves of the explant donor *C. canephora* and *C. arabica* were used as control for DNA ploidy level determination. The suspensions were analyzed with a Partec PAS^®^ cytometer (Partec^®^ GmbH, Münster, Germany).

In addition, roots were excised and treated according to Sanglard et al. (2017) to determine the 2n chromosome number of the plantlets previously screened by flow cytometry. From these roots, we prepared slides by cell dissociation and air-drying. All slides were stained with 5% Giemsa for 20 min, washed two times in dH_2_O and analyzed under a Nikon Eclipse Ci-S microscope (Nikon). Prometaphases and metaphases were captured using 100x objective and a CCD camera (Nikon Evolution™) coupled to a Nikon 80i microscope (Nikon).

### DNA Sequence Stability and 5-Methylcytosine Level

Due to different responses obtained during the somatic embryo recovery in M3 and M4, DNA sequence stability (SSR markers) and 5-methylcytosine level (5-mC%) were evaluated in order to identify genetic (SSR markers) and epigenetic (5-mC%) differences related to *in vitro* responses. For this, besides of leaf of the explant donor plants, the following DNA samples of the cellular mass were used: (a) not colchicine-exposed (control – friable calli of the M1), (b) colchicine-exposed and without somatic embryos, and (c) colchicine-exposed and with MCSE, totalling to 32 samples for DNA sequence stability and 21 for 5-mC% analyses (**Table S1**). Because at least 30 µg of genomic DNA are necessary to accomplish the 5-mC% measurement, cellular masses of the same treatment and *in vitro* response (a, b, or c—above) were put together. Genomic DNA was extracted from the explant donor *Coffea* plants and the cellular mass and macerated in MagNALyser (Roche^®^, Germany) for 70 s at 6,300 rpm (Doyle and Doyle, 1990), with addition of 7.5 M C_2_H_3_O_2_NH_4_ and excluding the overnight period for DNA precipitation. DNA purity and concentration were estimated using NanoDrop (Thermo Scientific^®^ 2000c).

DNA sequence stability was evaluated employing ten SSR primers (SSR Ca002, SSRCa021, SSRCa045, SSRCa091, SSRCa006, SSRCa084, SSRCa085, SSRCa087, SSRCa088, and SSRCa095) developed and validated for *C. arabica* (Missio et al., 2009). PCR reactions were performed in a final volume of 15 µL composed of: 3 µL of 5×buffer, 1.5 mM of dNTPs, 0.2 µM of primers, 50 ng of DNA, 1.6 mM of MgCl_2_, 1 U of Taq DNA polymerase, and sufficient quantity of dH_2_O to 15 µl. Amplifications were carried out in a Bio-Rad^®^ 96-Well Thermal Cycler C1000™ by touchdown PCR procedure, as performed by Sanglard et al. (2017) for allotriploid and hexaploid “Híbrido de Timor”. PCR products were submitted to electrophoresis on 10% polyacrylamide gel in 1X TBE buffer for 4 h at 100 V. The gels were stained with ethidium bromide solution (0.25 mg ml^-1^) for 20 min, and photo-documented in a Bio-Rad Molecular Imager^®^ Gel Doc ™ using the Image Lab program. The allele forms were tabulated considering the number and position of the bands.

Global 5-mC% was measured through high-performance liquid chromatography (HPLC) using 30 µg of DNA diluted in sterile dH_2_O for 100 µl of solution. DNA samples were hydrolyzed with 50 µl of 70% perchloric acid at 100°C for 1 h and the pH 4 (Chen et al., 2013). The solutions were analyzed in Prominence HPLC (Shimadzu^®^, Japan). The global 5-mC% of each sample was determined by comparison with standards of cytosine (C) and 5-mC for HPLC (Sigma^®^). The global 5-mC% in the DNA was calculated by %5-mC = [5-mC/(C + 5-mC)] × 100.

### Statistical Analysis

The total number of MCSE was compared by *F* test (*P* ≤ 0.05) and represented in graphics. Cellular masses [(a) not colchicine-exposed (control–friable calli of the M1), (b) colchicine-exposed and without somatic embryos and (c) colchicine-exposed and with MCSE] were compared in relation to their DNA sequence stability and their global 5-mC%. A contingency table was generated considering all allele forms found for each SSR primer and for each defined cellular mass. After analysis of variance (ANOVA), we performed a correspondence analysis from the contingency table to verify the relation between the allele forms and cellular masses. The mean global 5-mC% values were compared by ANOVA, followed by Dunnett’s test (*P ≤* 0.05). Statistical comparisons were performed using the software R (R CORE TEAM, Version 3.1.1, 2014-07-10).

## Results

### Indirect Somatic Embryogenesis Response

After 90 days in M1 (**Table 1**), the mean number of responsive explants, which were defined by leave fragments with friable calli, was 1.07 for *C. canephora* and 2.37 for *C. arabica*. In M2 (**Table 1**), CAS were established from friable calli of the two *Coffea* species after the third subculture, equivalent to 45 days. So, the CSD procedure was conducted in the seventh day during the fourth subculture. After transferring the cellular aggregates to M3 or M4, they were denominated cellular mass (**Figure 1**).

**Figure 2:**
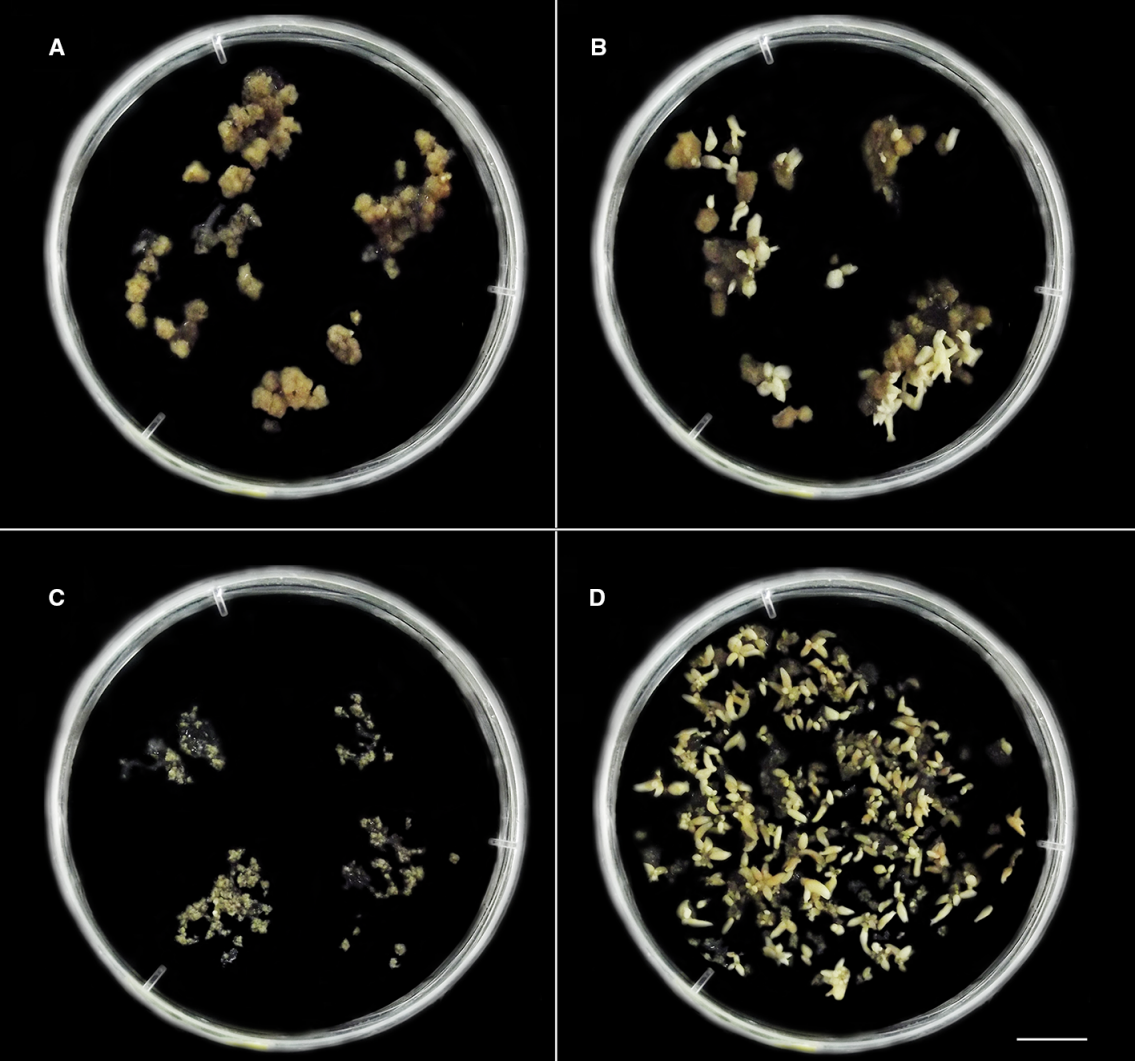
The distinct *in vitro* responses from cellular mass of *C. canephora* **(A, B)** and *C. arabica* **(C**, **D)** observed after colchicine treatment in liquid medium M2 (**Table 1**, **Figure 1**). **(A, B)** *C. canephora* cellular mass after 150 days (**Table 2**) treated with 2.5 mM/48 h colchicine (**Figure 1**) and maintained in M4 medium (**Table 1**). **(A)** Petri dish showing predominance of unresponsive cellular mass and others with few globular somatic embryos (spotlight – right/above). **(B)** Petri dish exhibiting one unresponsive cellular mass and all others with somatic embryos in distinct development stages (globular, heart, torpedo, and cotyledonary). **(C)** Unresponsive cellular mass of *C. arabica* treated with 1.5 mM/72 h colchicine and maintained in M4 during 90 days. **(D)** Several *C. arabica* somatic embryos in different development stages. This result was obtained after 90 days from CAS treated with 0.5 mM/72 h colchicine and the resulting cellular mass maintained in M4. Bar = 1 cm.

**Figure 3:**
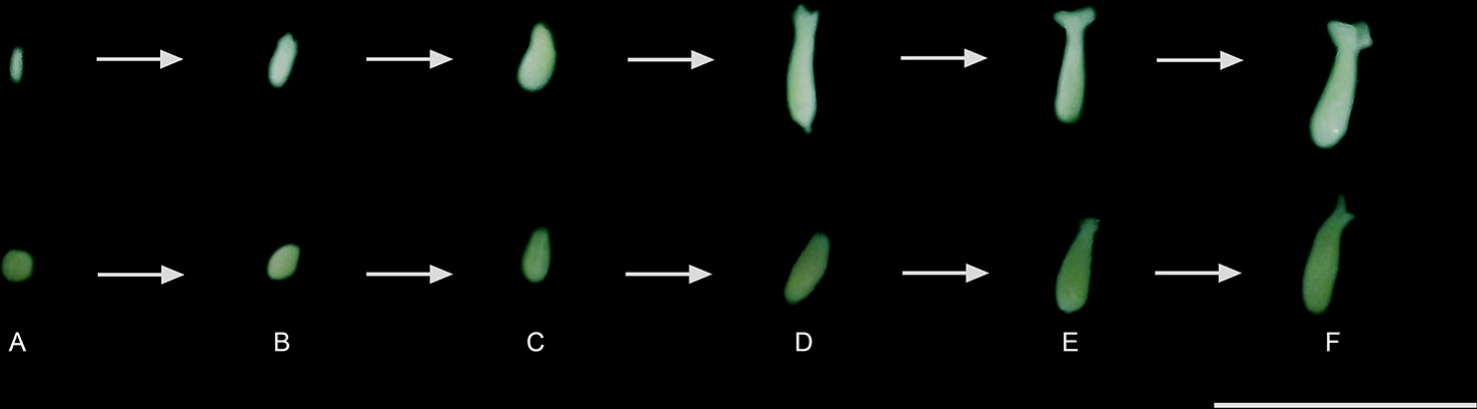
Regeneration, conversion, and maturation of the somatic embryos of *C. canephora* and *C. arabica*: **(A)** globular, **(B)** heart, **(C)** torpedo, **(D)** initial, **(E)** middle, and **(F)** mature cotyledonary. The MCSE **(F)** were transferred to M5 (**Table 1**) and, thus, considered for comparison of the treatments. Bar = 1 cm.

*C. canephora* somatic embryos were regenerated from cellular mass treated with 2.5 mM/48 h colchicine and maintained in M3 or M4 and 2.5 mM/72 h colchicine in M3. For *C. arabica,* somatic embryos were recovered only from 0.5 mM/72 h colchicine in M3 or M4 (**Figure 2**). The cellular mass of these *Coffea* species presented globular somatic embryos (**Figure 3A**), which were converted in heart (**Figure 3B**), torpedo (**Figure 3C**) and cotyledonary stages (**Figures 3D–F**). The cotyledonary somatic embryos matured into MCSE (**Figure 3F**). Somatic embryos in different development stages were recorded in the same responsive cellular mass, evidencing an asynchronized ISE response (**Figures 2** and **3**). The responsive cellular masses were statistically identical in relation to somatic embryo number.

In M3 and M4, we recovered 324 MCSE for *C. arabica* after 90 days and 76 for *C. canephora* after 120 days (**Table 2**, **Figure S1**). Thus, the regeneration response occurred in different moments for the two *Coffea* species, the colchicine treatments (time and concentration) and for M3 and M4 media. We counted a total of 878 MSCE in the experiment, of which 621 were from *C. arabica* and 257 from *C. canephora*. The plantlets were recovered gradually from the MCSE in M5 medium (**Table 1**) after 60 days, resulting in 392 (44.6%) plantlets out of which 103 plantlets belonged to *C. canephora* and 289 to *C. arabica* (**Table 1**). This reduced number of plantlets in relation to MCSE is due to failure or inadequate morphological development of the root and shoot. The cellular masses showed an almost continuous production of somatic embryos for both species, over a time span of more than one year, with potential for plantlet recovery over several months. However, we only regarded embryos until 150 days in M3 or M4 in this study.

**Table 2:** Number of recovered MCSE, number (%) of plantlets regenerated, and number (%) of autotetraploid *C*. *canephora* and auto-alloctaploid *C. arabica* regenerated from the CSD procedure (**Figure 1**).

*Coffea* species	Colchicine treatment	Medium^1^	MCSE in 90 days^2^	MCSE in 120 days^2^	MCSE in 150 days^2^	Total of MCSE	Total of plantlets^3^	Total (%) of plantlets with chromosome set doubling^4^
*C. canephora*	2.5 mM/48 h	M3	–	25	100	125	45 (36.0%)	12 (26.7%)
		M4	–	29	70	99	37 (37.4%)	16 (43.2%)
	2.5 mM/72 h	M3	–	22	11	33	21 (63.6%)	8 (38.1%)
				76	181	257	103 (40.1%)	36 (34.9%)
*C. arabica*	0.5 mM/72 h	M3	89	70	78	237	102 (43.0%)	27 (26.5%)
		M4	235	40	109	384	187 (48.7%)	34 (18.2%)
			324	110	187	621	289 (46.5%)	61 (21.1%)

1—**Table 1**.

2—Total number and % of MCSE regenerated in M3 or M4 and transferred to M5 (**Table 1**).

3—Total number and % of plantlets showing leaves for DNA ploidy level assessment after two months in M5.

4—Number and % of plantlets that showed the DNA ploidy level equivalent to the double in relation to the respective explant donor plant.

### Plantlet Screening for Chromosome Set Doubling

**Figure 4:**
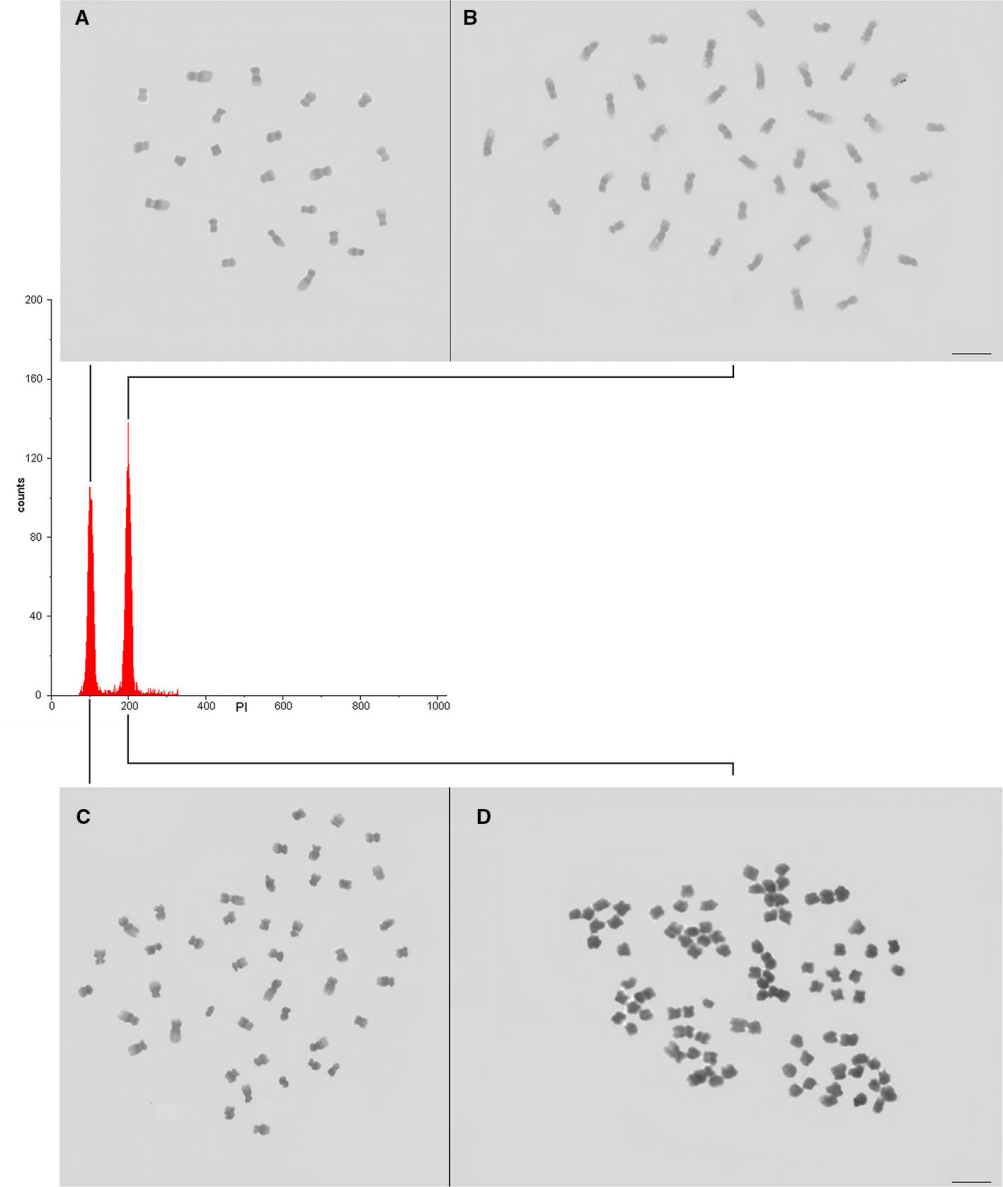
Confirming the CSD of the *Coffea* CAS. **(A, B)** Nuclear DNA content and ploidy level of the diploid (**A**—explant donor plant, 2C = 1.43 pg, 2x = 22 chromosomes) and autotetraploid (**B**—4C = 2.86 pg, 4x = 44 chromosomes) *C. canephora*, and **(C, D)** tetraploid (**C**—explant donor plant, 2C = 2.62 pg, 4x = 44 chromosomes) and auto-alloctaploid (**D** – 4C = 5.24 pg, 8x = 88 chromosomes) *C. arabica*. As the DNA ploidy level determination and nuclear genome size measurement were separately accomplished for *C. canephora* and for *C. arabica*, the channel of the internal standard (explant donor plant) G_0_/G_1_ nuclei peak was adjusted to 100. So, the G_0_/G_1_ nuclei peak of the autotetraploid *C. canephora* and auto-alloctaploid *C. arabica* occurred in channel 200.

DNA ploidy level was determined for individual plantlet (392 plantlets, **Table 2**, **Figure S1**) upon comparison with the G_0_/G_1_ nuclei peak of the explant donor *Coffea* plant (**Figure 4**). For the recovered plantlets screened for CSD, the DNA ploidy level was newly determined after two months and after six months. So, the polyploid condition was verified for all *Coffea* plantlets (**Table 2**) in all flow cytometry analyses, confirming the solid polyploid condition. We found no mixoploid plantlets.

We screened plantlets with a DNA ploidy level equivalent to tetraploidy (4C = 2.86 ± 0.053 pg) for *C. canephora* (explant donor plant with 2C = 1.43 pg, 2n = 2x = 22 chromosomes), being 12 plantlets (26.7%) from 2.5 mM/48 h/M3, 16 (43.2%) from 2.5 mM/48 h/M4, and 8 (38.1%) from 2.5 mM/72 h/M3. So, we found a total of 36 (34.9%) tetraploid plantlets for *C. canephora* (**Table 2**). The tetraploidy of these plantlets was confirmed by chromosome counting, showing 2n = 4x = 44 chromosomes. Therefore, these new germplasms represent *C. canephora* autotetraploids (**Figure 4**).

The CSD procedure (**Figure 1**) also provided octaploid plantlets of *C. arabica* (explant donor plant with 2C = 2.62 pg, 2n = 4x = 44 chromosomes). These plantlets, with 4C = 5.24 ± 0.028 pg and 2n = 8x = 88 chromosomes (**Figure 4**), were obtained from 0.5 mM/72 h/M3 (27 plantlets—26.5%) and M4 (34 plantlets—18.2%). Thus, we screened a total of 61 (21.1%) plantlets as octaploid for *C. arabica* (**Table 2**). Considering the evolutive origin of this species, which is a true allotetraploid from *C. canephora x C. eugenioides*, and the ploidy level of the plantlets (4C = 5.24 pg and 2n = 8x = 88), these germplasms can be considered as auto-alloctaploid.

No plantlets were recovered from the cellular masses that were not colchicine-treated or from other colchicine treatments. Due to these different *in vitro* responses (friable calli without MCSE and friable calli with MCSE), we evaluated genetic stability (using SSR markers) and global 5-mC% to verify if these differences are associated with DNA sequence (genetic stability) and/or 5-mC% level (epigenetic) changes. Henceforth, the recovered somatic embryos were used as a parameter to define the responsiveness of the cellular masses.

### DNA Sequence Stability and 5-Methylcytosine Level

The ten selected SSR primers, which were developed for *C. arabica*, amplified DNA sequences for the two *Coffea* explant donors and for all sampled cellular masses. One to six allelic forms were evidenced from the ten SSR primers. The primer SSRCa006 was monomorphic for the explant donor plants and all cellular masses for two *Coffea* species. The primer SSRCa091 was monomorphic only for *C. arabica*. The primers SSRCa084 and SSRCa091 were monomorphic for the responsive and non-responsive cellular masses treated with colchicine in *C. canephora* and primer SSRCa085 in *C. arabica*. Specifically, for *C. arabica*, only one cellular mass showed a different allelic form for primers SSRCa085, SSRCa088 and SSRCa095.

The primer SSRCa002 was the most polymorphic, evidencing six allelic forms for *C. canephora* and five for *C. arabica*. This primer amplified three allelic forms in not colchicine-treated cellular mass (friable calli of the M1) of *C. canephora*, and five in cellular mass treated with 2.5 mM colchicine/48 h and maintained in M3 and M4. For *C. arabica*, the same primer provided two alleles for not colchicine-treated cellular mass (friable calli of the M1), and three for the other cellular masses. This shows that for this primer, as well as for other primers, some alleles were specific for each *Coffea* species and for different cellular masses. Despite of the observed polymorphisms, there was no significant difference among the DNA sequences of the *Coffea* explant donors and the cellular masses, colchicine-treated or not, and with or without MCSE.

From these SSR polymorphisms, it was possible to verify that some allele forms were more common for the cellular mass with or without MCSE. The association of the found alleles with cellular masses was identified from the exploratory and descriptive correspondence statistical analysis. SSR045 allele 2 (SSR045_2), SSR002_6, SSR087_45, SSR088_2 were correlated to cellular mass with MCSE, and SSR045_3, SSR002_4 and SSR095_2 with cellular mass without MCSE. Therefore, the somaclonal variation occurred at DNA sequence level, which is demonstrated by emergence and disappearance of alleles verified in some sampled cellular masses, especially for the primers SSRCa002 and SSRCa045. However, its rates are not significant and do not explain the different responses of ISE among the cellular masses.

In the comparison of the mean values of global 5-mC% between *C. canephora* and *C. arabica* explant donor plants with the cellular masses, we were able to identify three different groups using Dunnett’s test. In the first group, the mean 5-mC% values of the donor explant plants were statistically identical with 18.33% of *C. canephora* and 18.00% of *C. arabica*. In the second group, global methylation levels were slightly reduced to 13.41% for *C. canephora* and 14.45% for *C. arabica* in the friable calli developed in M1 medium. In the third group, the global 5-mC% was higher in the cellular masses than in the explant donor plants during somatic embryo recovering in M3 or M4, after colchicine treatments: 23.56% for *C. canephora*/2.5 mM/48 h/M3 or M4 without MCSE, 25.29% for *C. canephora*/2.5 mM/48 h/M3 or M4 with MCSE, 25.24% for *C. arabica*/1.5 mM/72 h/M3 or M4 without MCSE, 26.23% for *C. arabica/*0.5 mM/72 h/M3 with MCSE, and 29.13% for *C. arabica*/0.5 mM/72 h/M4 with MCSE. Among them, the *C. arabica*/0.5 mM/72 h/M4 showed the highest mean value of global 5-mC% (**Table S1**).

## Discussion

In this study, a novel CSD procedure was established for two *Coffea* crop species from CAS of the diploid *C. canephora* and the true allotetraploid *C. arabica*, resulting in solid autotetraploid *C. canephora* (2n = 4x) and auto-alloctaploid *C. arabica* (2n = 8x) plantlets. These new germplasms were approximately regenerated within one year: 90 days for friable calli production, 67 days for CAS establishment, 2–4 days for colchicine treatment, 150 days for MCSE regeneration, and 60 days for plantlet recovery. This relative short time represents an advance in *Coffea* breeding programs, which depend on strategies that demand several crossings, large progeny and a long time to provide new germplasms. For instance, the *in vitro* hybrid selection time (~8 years) is shorter than the hybrid selection time (~25 years) in the traditional breeding program (Etienne et al., 2018). In addition, the new germplasms were formed from a simple and small leave fragment of the selected plants. *Coffea* leave cells showed a higher degree of plasticity, allowing them to reprogram and to form the somatic embryo and the plantlet from the following ISE *in vitro* morphogenic pathway: differentiated → dedifferentiated → redifferentiated.

To induce the polyploidization in liquid system, we explored the high proliferation of the *Coffea* CAS (van Boxtel and Berthouly, 1996; Clarindo et al., 2012). CAS are maintained in constant orbital agitation, allowing that more cells have direct contact with the compounds of the medium, mainly the growth regulators that promote cell proliferation (2,4-D in our study) and the antitubulin (here, the colchicine) during the CSD procedure, than in semisolid system (Loyola-Vargas and Vázquez-Flota, 2006; Dutt et al., 2010). In semisolid system, there is, for example, a reduction in the rate of nutrient diffusion due to the gelatinous consistency of the medium (Loyola-Vargas and Vázquez-Flota, 2006).

The key issue of the procedure (**Figure 1**) was the antitubulin treatment applied to proliferating CAS in liquid *in vitro* system. Again, the chemical and physical conditions of the tissue culture environment were effective, giving rise to friable calli (first step—dedifferentiation) and CAS with proliferative cells (second step), MCSE (third step) and plantlets with different ploidy level (fourth step). This ISE pathway has been extensively exploited for *Coffea* plantlet regeneration since van Boxtel and Berthouly (1996). In addition, it was reproducible for different species and genotypes of the same species (Samson et al., 2006; Almeida et al., 2008; Ibrahim et al., 2015), such as the diploid *C. canephora* and *C. eugenioides*, allotriploid “Híbrido de Timor” “CIFC 4106” and true allotetraploid *C. arabica* (Sanglard et al., 2019). Therefore, the obtained new *Coffea* germplasms are a statement that van Boxtel and Berthouly’s (1996) *in vitro* conditions are the base to establish tissue culture protocols for this genus.

*Coffea* MCSE recovery occurred after removing the exogenous auxin 2,4-D and supplementing the tissue culture medium with activated charcoal. Thus, the elimination of the main chemical component responsible for keeping the cells in totipotent condition was required, as recommended by Rose et al. (2010) and Nic-Can and Loyola-Vargas (2016). Activated charcoal is added to tissue culture because of its adsorption capacity of exogenous 2,4-D residues (Pan and van Staden, 1998) and by adsorbing medium-inhibiting substances or toxic products released by cells, helping to promote the somatic embryo regeneration, conversion and maturation to mature cotyledonary. The osmotic control also is fundamental for somatic embryo recovery. For friable calli induction, CAS establishment and proliferation, a relatively high osmotic potential was necessary. On the order hand, the decrease of osmotic potential is fundamental for somatic embryo regeneration, mimetizing the seed environment during the zygotic embryo development (Dutt et al., 2010).

**Figure 5:**
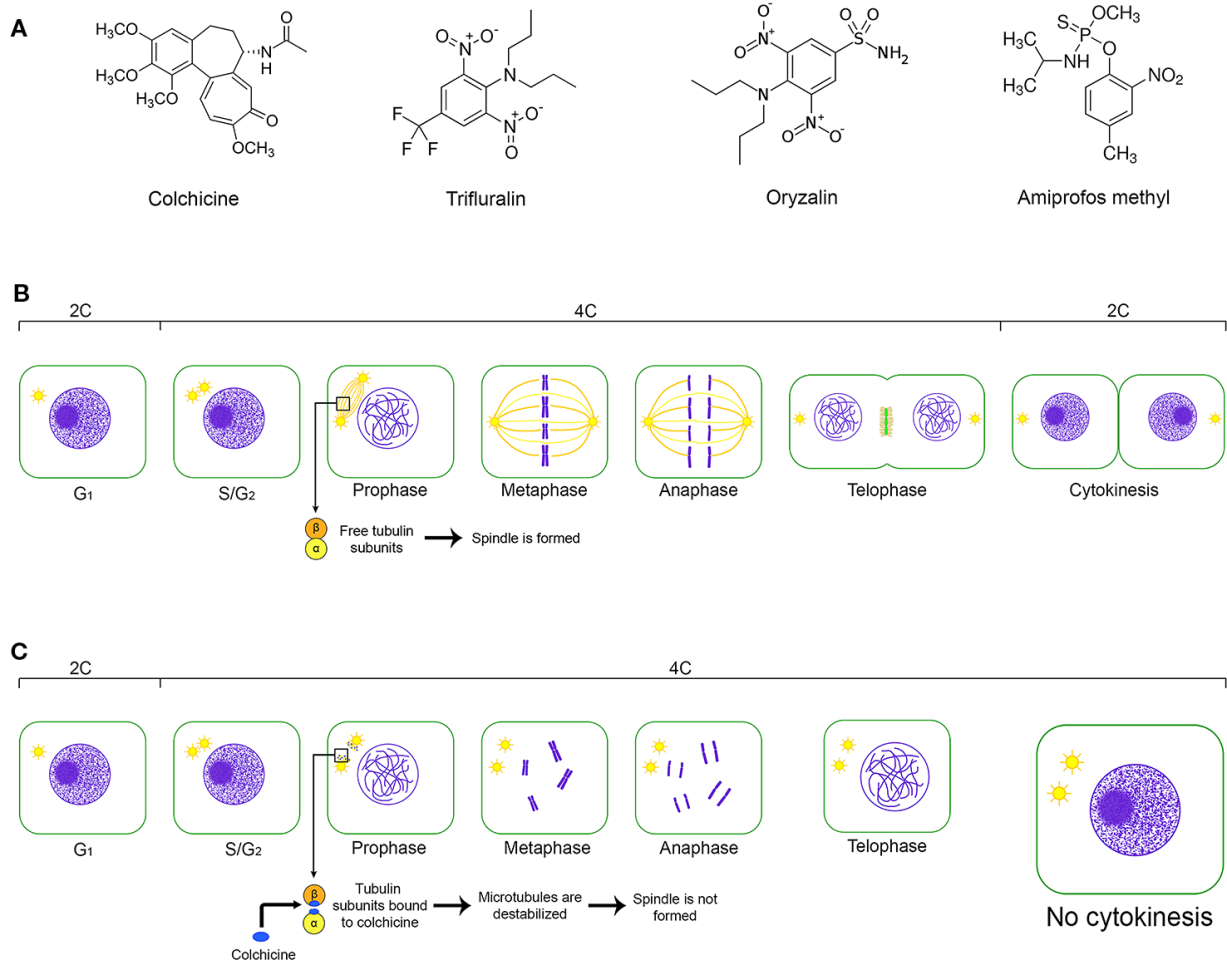
The key issues of the CSD from a cell of the friable calli. **(A)** Chemical structure of the main antitubulin compounds used for CSD: colchicine—(S)-*N*-(5,6,7,9-tetrahydro-1,2,3,10-tetramethoxy-9-oxobenzo[a]heptalen-7-yl)acetamide; oryzalin—3,5-dinitro-N*^4^*,N*^4^*-dipropylsulfanilamide; trifluralin α,α,α-trifluoro-2,6-dinitro-*N*,*N*,-dipropyl-*p*-toluidine; and amiprofos methyl—O-methyl O-(2-nitro-*p*-tolyl) N-isopropylphosphoramidothionate. **(B)** Representation of the cell cycle without the interference of an antitubulinic agent, its cellular events and the changes of the relative DNA content (2C, 4C) considering a diploid cell in G_1_. **(C)** The effect of the antitubulin agent on the cell cycle and, consequently, on the relative DNA content. Antitubulin agents prevent the mitotic fusion formation, avoiding the chromatide segregation and cytokynesis occurence. So, the chromosome set is duplicated in the same cell nuclei, which shows relative DNA content equivalent to 4C in G_1_.

As summarized in **Figure 5**, the principle to promote the CSD was to polyploidize as many cells as possible of the *Coffea* CAS using the antitubulin colchicine, which may be replaced by another compound with the same and specific effect. The very specific (Planchais et al., 2000) and cytotoxic (Dutt et al., 2010; Acanda et al., 2015) antitubulin compounds hinder mitotic fuse formation, which is fundamental for chromatid segregation during anaphase and for cytokinesis. Considering this as well as the ploidy level, the number of chromosome sets in both *Coffea* species was duplicated due to the action of the anaphase promoting complex (APC)-activated separase pathway and prevention of cytokinesis. Separase (Esp1), a cysteine protease, cleaves the SCC1 subunit of the cohesin (Orr-Weaver, 1999; Tanaka et al., 1999), a protein complex constituted by conserved polypeptides SMC1, SMC3, SCC1 and SCC3 (Cai et al., 2003). This protein keeps the sister chromatids together (Orr-Weaver, 1999; Tanaka et al., 1999) from S-phase (interphase) to initial anaphase. As the polyploidization occurred in *Coffea* CAS, we concluded that the cohesin was cleaved, doubling the chromosome set number as in a normal anaphase. However, these chromosomes remained in the cell as a result of the absence of the mitotic fuse and, consequently, cytokinesis non-occurrence. The nuclear membrane reorganization around these chromosomes happens in telophase, and daughter cells were formed with one nucleus in polyploid condition in comparison to the *Coffea* donor plant.

The colchicine application on the seventh day after the subculture also contributed to the generation of 36 (34.9%) autotetraploid *C. canephora* and 61 (21.1%) auto-alloctaploid *C. arabica*. For *Citrus*, Dutt et al. (2010) also treated the suspension cells with colchicine after seven days in the third subculture. Our results, as well as Dutt et al. (2010) and Zhang et al. (2007), highlight that the moment for antitubulin treatment should be chosen according to the number of cells in S-phase (interphase). This can be checked for each plant species using flow cytometry, for *Coffea*, this cell cycle phase occurs after 7 to 9 days (van Boxtel and Berthouly, 1996). In addition, this previous data increases experimental control and, consequently, the number of polyploids because it allows to attest that the cells are proliferative, to choose a day before mitosis and cytokinesis, and to conduct colchicine treatment in a shorter time.

Another impact of the CSD procedure was the recovery of pure polyploids of the both *Coffea* species. This result corroborates with the ISE possibility of regeneration of the somatic embryo and, consequently, a plantlet from only one cell of the friable calli (semisolid system, (Wu and Mooney, 2002; Petersen et al., 2003; Zhang et al., 2007; Sanglard et al., 2017) or CAS (liquid system, Dutt et al., 2010; Acanda et al., 2015). Moreover, CSD using proembryogenic cells of friable calli or CAS reduced or nulled the regeneration of mixoploid plantlets (Dutt et al., 2010; Acanda et al., 2015; Sanglard et al., 2017). Besides, CAS are considered meristematic cells, becoming a successful source for CSD and providing solid polyploids. Therefore, the CAS can replace the shoot meristems in buds generally used for CSD (Dhooghe et al., 2011). CSD from the shoot apical meristem demands the polyploidization of all, or at least most, cells that constituted the periphery, central and medullar zones (L1, L2, and L3 layers). Therefore, mixoploids generated from this explant are result of the failure of the CSD.

Although the ISE is an advantageous system for CSD (Wu and Mooney, 2002; Petersen et al., 2003; Zhang et al., 2007; Sanglard et al., 2017), we also recorded cellular masses without somatic embryos and another with continuous somatic embryo regeneration, conversion and maturation. The first hypothesis to explain these ISE response divergences is the cytotoxic effect of the colchicine, but the non-colchicine treated cellular masses did not show somatic embryos. Besides, the responsive cellular masses of *C. canephora* were treated with 2.5 mM colchicine, exactly the highest concentration of this compound. Acanda et al. (2015) related that the colchicine exposure reduced the embryogenic potential in comparison to the control. The same has been reported by several authors independently for biological material used for *in vitro* CSD.

Another possible explanation is the occurrence of somaclonal variation (SV), which is a common phenomenon in cell culture caused by genetic and epigenetic changes in the nuclear genome, as well as genetic changes in organelle genomes (Kaeppler et al., 2000; Wang and Wang, 2012). Therefore, the term SV describes the variability produced by *in vitro* propagation due to physical and chemical conditions, time, and employed propagation system (liquid or semisolid). To test the hypothesis that SV occurred, we used SSR DNA markers to monitor cellular masses. We observed clonal fidelity in relation to the *Coffea* explant donors and used molecular markers.

Global 5-mC% divergences were identified between the cellular masses in dependence of MCSE occurrence. *In vitro* plant cells were categorized in a genetic and evolutive context (Wang and Wang, 2012) according to the level and effect of the SV: true cells, neutral cells, deleterious cells and beneficial cells. The term true cell is designated for the *in vitro* cell without SV, but with the possibility of epigenetic modifications essential for the morphogenic process. A neutral cell is defined as the *in vitro* cell with SV, but having the same phenotype as the ancestral cell without changes in fitness. Neutral cells also include cells with deleterious SV but suppressed by other genome changes or by suppressor genes. Deleterious cells are defined as the *in vitro* cells containing SV, which leads to a decrease in the fitness relative to ancestral cell. Eventually, the beneficial cell is a cell exhibiting SV that increases the fitness value compared to ancestral cells. Based on these concepts by Wang and Wangs’ (2012) and in our results, we concluded that the cellular masses of both *Coffea* species showed true and neutral cells. However, other aspects of the genome (as other molecular markers), epigenome (as the histone chemical change and 5-mC% of the genes), transcriptome and metabolome should be evaluated in future studies in order to understand the factors that have hindered the somatic embryo generation.

## Conclusion

Our results suggest that the CSD using ISE and the antitubulin treatment of the CAS is a successful procedure to produce solid polyploid plants of *Coffea* and potentially for other species as well. We generated new *Coffea* germplasm, autotetraploid *C. canephora* and auto-alloctaploid *C. arabica*, that constituted a diversification of the *in vitro* germplasm of this genus. These individuals can be used as explant donor for other tissue culture procedures or be acclimatized for morphologic, physiologic and reproductive evaluations. Besides, the epigenetic modulation of the chromatin was associated to somatic embryo regeneration and, consequently, to plantlet recovery. Therefore, the tissue culture conditions should promote this typical change related to cellular redifferentiation.

## Data Availability Statement

The raw data supporting the conclusions of this article will be made available by the authors, without undue reservation, to any qualified researcher.

## Author Contributions

The authors LV, MM and WC conducted the tissue culture experiments and *in vitro* chromosome set doubling. WC carried out the cytogenetic and flow cytometry analyses. LV, PA-S and TS executed the SSR molecular analyses. LV, PA-S, and AP conducted epigenetic analyzes. LV, GC and AF did the statistical analysis. All authors equally contributed for manuscript editing and revision and approved the final manuscript for submission.

## Conflict of Interest

The authors declare that the research was conducted in the absence of any commercial or financial relationships that could be construed as a potential conflict of interest.

## Supplementary material

Table S1 Experimental design for DNA sequence variability and global DNA methylation level of individual cellular mass, and statistical comparison among the mean global 5-mC%.

Figure S1 Number of regenerated MCSE for *C. canephora* and *C. arabica* after 90, 120 and 150 days.
