# Supplementary material for: Autotetraploid Coffea canephora and Auto-Alloctaploid Coffea arabica From In Vitro Chromosome Set Doubling: New Germplasms for Coffea
Source: Front Plant Sci. 2020 Mar 4;11:154. doi: 10.3389/fpls.2020.00154 (PMC7064561; doi:10.3389/fpls.2020.00154)
Supplement: Table S1 — Experimental design for DNA sequence variability and global DNA methylation level of individual cellular mass, and statistical comparison among the mean global 5-mC%. [file Table_1.docx]

**Supplement 1** **–** Experimental design for DNA sequence variability and global DNA methylation level of individual cellular mass, and statistical comparison among the mean global 5-mC%.

| ***Coffea*** | **SSR markers** | |  | **HPLC** | | **Mean 5-mC%** |
| --- | --- | --- | --- | --- | --- | --- |
|  | **Number of samples** | **Biological material** |  | **Number of samples** | **Biological material** |  |
| *C. canephora* | 1 | Leaf of the plant explant donor |  | 1 | Leaf of the plant explant donor | 18.33%^c^ |
|  | 6 | Friable calli of the M1 |  | 6 | Friable calli of the M1 | 13.41%^d^ |
|  | 6 | 2.5 mM/48h/M3 or M4 without MCSE |  | 2 | 2.5 mM/48h/M3 or M4 without MCSE | 23.56%^b^ |
|  | 6 | 2.5 mM/48h/M3 or M4 with MCSE |  | 3 | 2.5 mM/48h/ M3 or M4 with MCSE | 25.29%^b^ |
| *C. arabica* | 1 | Leaf of the plant explant donor |  | 1 | Leaf of the plant explant donor | 18.00%^c^ |
|  | 6 | Friable calli of the M1 |  | 3 | Friable calli of the M1 | 14.45%^d^ |
|  | 2 | 0.5 mM/72h/M3 with MCSE |  | 2 | 0.5 mM/72h/M3 with MCSE | 26.23%^b^ |
|  | 2 | 0.5 mM/72h/M4 with MCSE |  | 1 | 0.5 mM/72h/M4 with MCSE | 29.13%^a^ |
|  | 1 | 1.5 mM/72h/M3 without MCSE |  | 2 | 1.5 mM/72h/M3 and M4 without MCSE | 25.24%^b^ |
|  | 1 | 1.5 mM/72h/M4 without MCSE |  |  |  |  |
| Total | 32 |  |  | 21 |  |  |

The mean global 5-mC% was compared by ANOVA followed by Dunnett’s test (*P ≤* 0.05).
