# Supplementary figures and images for: Autotetraploid Coffea canephora and Auto-Alloctaploid Coffea arabica From In Vitro Chromosome Set Doubling: New Germplasms for Coffea
Source: Front Plant Sci. 2020 Mar 4;11:154. doi: 10.3389/fpls.2020.00154 (PMC7064561; doi:10.3389/fpls.2020.00154)

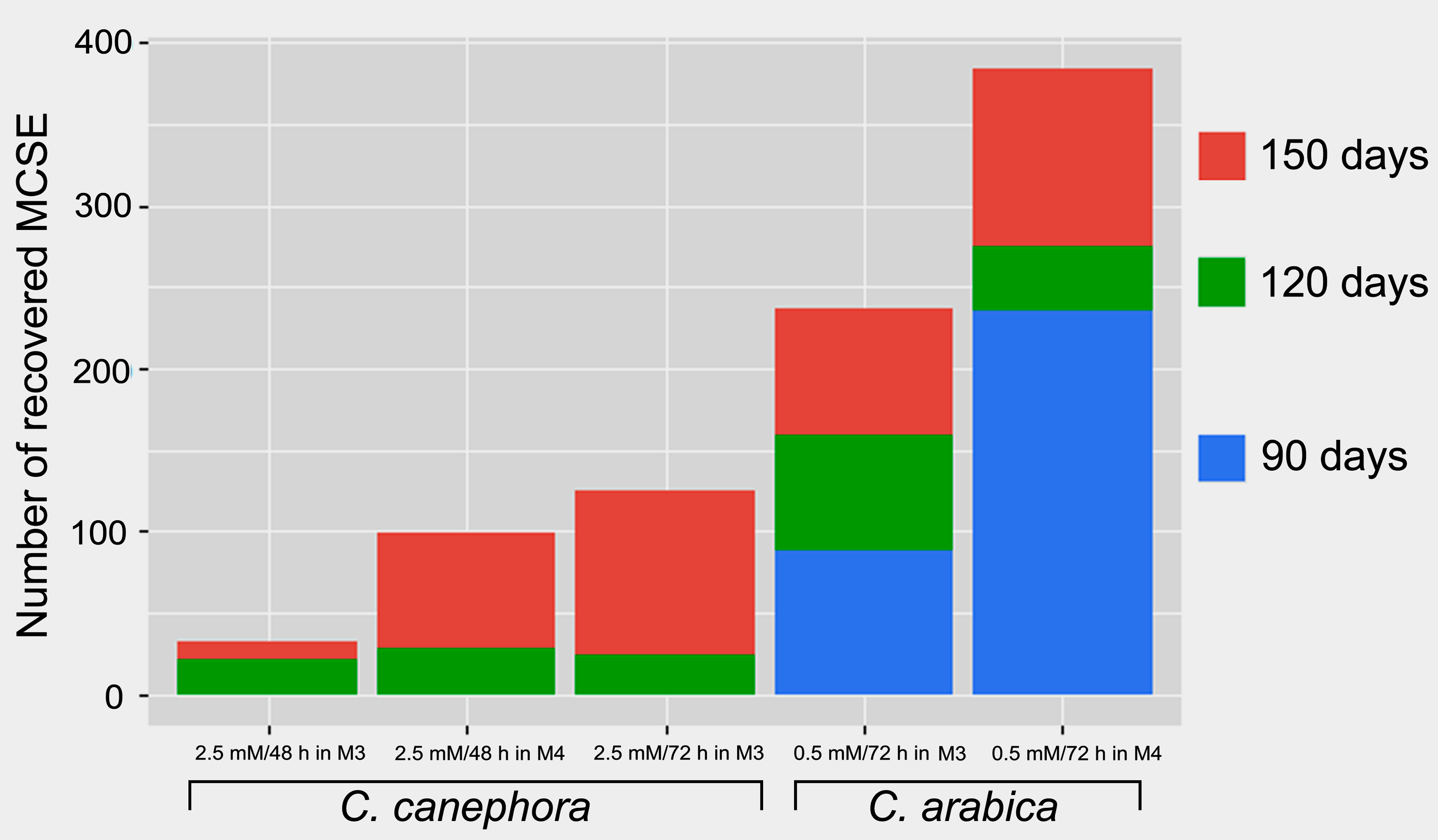

Supplement: Figure S1 — Number of regenerated MCSE for C. canephora and C. arabica after 90, 120 and 150 days. [file Image_1.tif]
